# Single coronary artery originating from the right sinus Valsalva and ability to work

**DOI:** 10.1186/s40557-015-0055-2

**Published:** 2015-01-24

**Authors:** Roberto De Rosa, Gennaro Ratti, Donato Gerardi, Carlo Tedeschi, Monica Lamberti

**Affiliations:** Department of Radiology, PSI ASL Napoli 1, (Via Ciccarelli 1), Naples, (80100) Italy; Department of Cardiology, San Giovanni Hospital, ASL Napoli 1, (Via Filippo Maria Briganti 255), Naples, (80100) Italy; Department of Experimental Medicine, Section of Hygiene, Occupational Medicine and Forensic Medicine, Second University of Naples, (Via dei Crecchio 16), Naples, (80100) Italy; Department of Cardiology, San Gennaro Hospital, ASL Napoli 1, (Via San Gennaro dei Poveri, 25), Naples, (80100) Italy

**Keywords:** Single coronary artery, Multi Slices Computed Tomography, Working capacity

## Abstract

We present a case of a 56-year-old male electrician who was admitted to the hospital with atrial fibrillation, atypical chest pain and dyspnea. He gave a history that on the morning he had working for almost 4 hours carrying out various activities with considerable physical effort. After cardioversion, conventional coronary angiography revealed a suspect of single coronary vessel (SCA) arising from the right sinus of Valsalva. The patient underwent multislice computed tomography that showed a SCA arising from the right sinus Valsalva and dividing in Right Coronary Artery (RCA) and Left Main coronary artery (LM). The finding of posterior course of the LM without atherosclerotic has proved crucial for the expression of an opinion of working capacity even with limitation.

## Background

The normal coronary circulation encompasses a wide spectrum of normal variants, which are often detected as incidental findings during coronary angiography (CAG). A single coronary artery ostium is a rare finding. In the general population, the incidence of single coronary artery is approximately 0.024% [1,2]. Clinically it may cause recurrent ischaemia, heart failure or sudden death. We introduce a case of an Journeyman electrician with average energy requirement for his working activity >6 Metabolic Equivalents of Task (METs), presenting with atrial fibrillation and chest pain after a morning of work.

## Case presentation

A 56-year-old male electrician, with treated hypertension (reported mean values of blood pressure: 130/95 mm Hg), was admitted with chest pain lasting for about 60 minutes. He was journeymen electrician specialized in commercial, residential and low-voltage wiring.

He gave a history that on the morning he had working for almost 4 hours carrying out various activities such as manipulate cables and wires through cavities or carry boxes of electrical equipment weighing about 20 kilos, with considerable physical effort.

**Figure 1:**
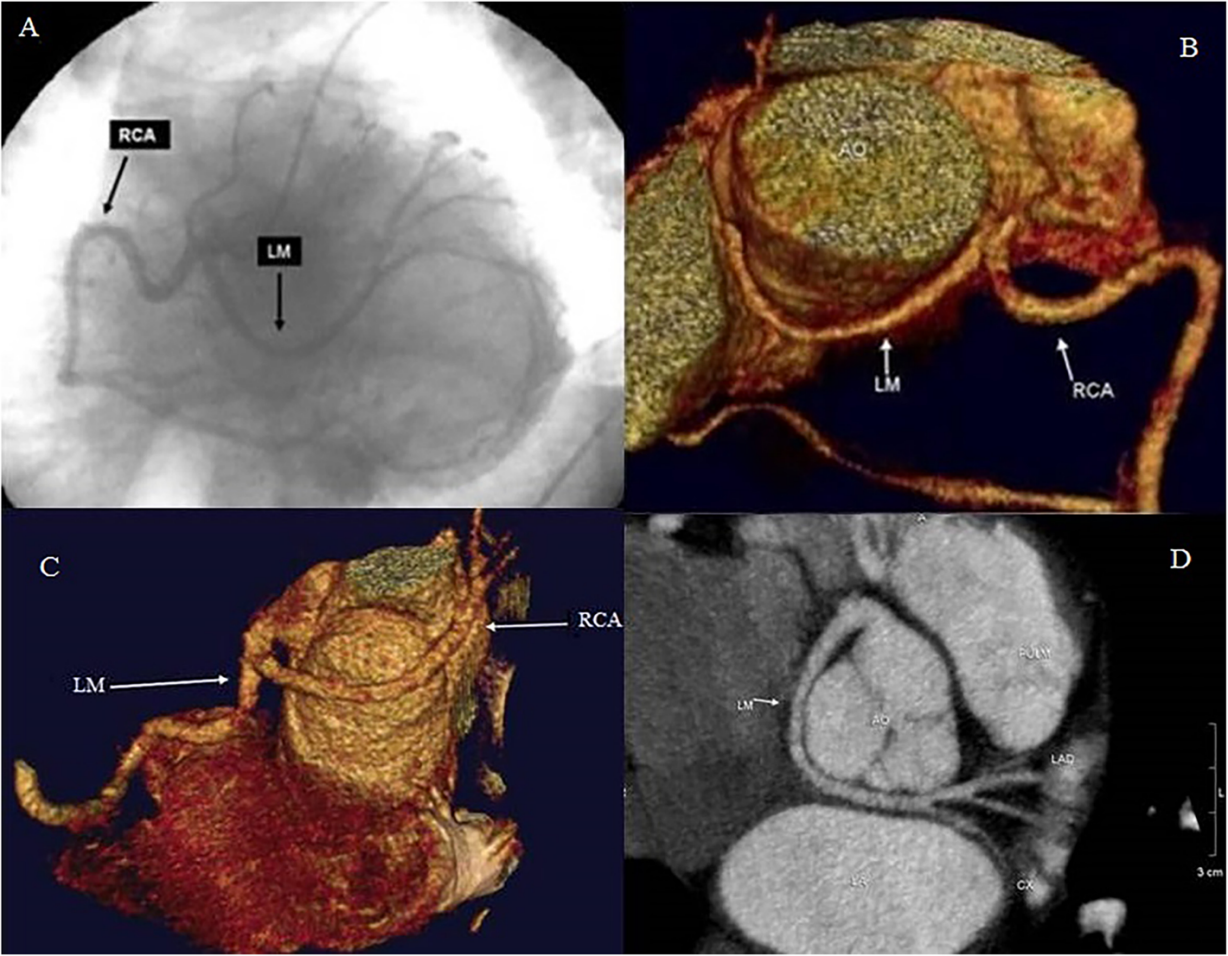
**Coronary angiography, volume rendering and curved multiplanar reconstruction from MSCT of the coronary anomalies. (Panel A)** Coronary Angiography (CAG) projection showing a Single Coronary Artery (SCA) arising from right sinus of Valsalva and dividing in right coronary artery (RCA) and Left Main (LM). Volume rendering **(panel B and C)** and Curved Multiplanar Reconstruction **(panel D)** from MSCT showed that LM turned posteriorly behind the aorta (non-malignant anomaly), reaching the atrio-ventricular groove where it divides into left anterior descending (LAD) and left circumflex (CX) arteries.

In his clinical history he reported to have had occasional atypical chest pain for which he submitted two years ago to exercise stress testing, which resulted maximal for age and negative for ischaemia induced by stress. The patient on admission had tachypnoea at rest with oxygen saturation of 93%. Electrocardiogram (ECG) showed atrial fibrillation with a heart rate of 90 beats/min. Blood pressure was 175/95 mm Hg. Cardiac auscultation was normal but at pulmonary level bilateral basal crackles were present. Cardiac enzymes were slightly elevated: Troponin T (TnT): 0,033 ng/l [norm: <0.03] and Creatine Kinase (CK): 260 U/l [norm: 47–220]. Regional left ventricular wall motion and ejection fraction was normal (Ejection Fraction EF 60%). After cardioversion with DC shock (3 J/Kg body weight), considering TnT and CK levels [3] a CAG was performed to exclude coronary disease. It revealed a suspect of single coronary vessel arising from the right sinus of Valsalva. The right coronary artery (RCA) showed a normal course (Figure 1: panel A). Stenoses of the coronary arteries were not present. In order to better define the origin and course of the single vessel, we performed a Multi Slices Computed Tomography (MSCT) (Figure 1: panels B, C and D) using a 64-detector-row scanner (Aquilion 64; Toshiba Medical System, Tokyo, Japan).

MSCT showed a SCA arising from the right sinus Valsalva and dividing in RCA and Left Main (LM) (Figure 1: panel B,C and D). The LM showed an acute take-off angle, turned posteriorly behind the aorta, reaching the atrio-ventricular groove where it divides into left anterior descending (LAD) and left circumflex (CX) arteries. No atherosclerotic plaque were present. The patient was discharged with only antihypertensive drug therapy. After 2 month, at follow up medical check, he reported no angina and no exertional dyspnea. His left ventricular ejection fraction was normal and exercise tolerance >12 METs. He returned to his full work duties, although we recommended him to avoiding strenuous work (under 6 METs) and work on overhead lines too.

## Discussion

Evaluating the capacity of a patient with coronary artery anomalies to carry out occupational work often poses a difficult problem for the physician. The reason for the difficulty is that many factors influence any recommendation regarding these patients’ return to work, and specific guidelines for these condition, due to the limited number of affected patients in working age, have not been critically evaluated.

In fact the overall incidence of coronary anomalies in humans is 0.6 to 1.3% out of which the single coronary artery originating from the right coronary sinus accounts for 1.3% of all coronary anomalies [4-7]. Concerning the course of a single coronary vessel arising from the right coronary sinus, four anatomical variants have been described according to the crossing of the LM to the left side of the heart: (1.) anterior: LM turns anteriorly in front of the right ventricular outflow tract; (2.) inter-arterial: LM lies between the great vessels, aorta and pulmonary artery; (3.) septal: LM has an intramyocardial septal course; (4.) posterior: LM turns posteriorly behind the aorta in infero-posterior direction [8-10]. The inter-arterial course has been known as cause of sudden cardiac death (>50%) [11]. Other types can present with myocardial ischaemia, congestive heart failure and sudden cardiac death. Myocardial ischaemia is thought to occur due to the impaired coronary flow reserve and also secondary to the anatomic and functional anomaly of the ostium (acute aortocoronary angulation, slit-like ostium, ostial tissue flaps and initial course of the coronary artery within the aortic wall) [8]. In the inter-arterial variant, ischaemia or sudden death are assumed to be caused by vascular compression or kinking [8,11,12]. Our case was the posterior variant judged as non-malignant anomaly without remarkable changes in cardiovascular risk [8,10]. It is very important to define the course of the coronary anomaly because some misinterpretations of the coronary angiography are possible. Therefore, additional nonivasive imaging methods with better spatial resolution like MSCT have been suggested [4,13-16]. Since the overall number of patients is small, the treatment strategy varies and is not clearly defined. Generally when an adult or older patient is incidentally diagnosed wrong sinus coronary malformations, in absence of coronary artery disease and in consideration that sudden death occurs at a young age, this occasional finding has likely no clinical significance and most probably needs no surgical therapy [17-19]. Nevertheless when these subjects are symptomatic, a clear guidelines have not yet been defined and the treatment is still controversial. The finding, in our case, of posterior course of the LM without atherosclerotic lesions has proved crucial to determine the therapeutic treatment with only antihypertensive drugs and to allow the patient to re-entry into the workforce even with suggestion to reduce the effort under 6 METs (as the transport of weights above 15 kilos) and to avoiding work on overhead lines too, although the patient reported that he had never worked on power lines [20,21].

In fact, in our case, although the anatomical variant has been classified as benign, the non-certain cause of high level of TnT and CK and the presence of atrial fibrillation after a strenuous work, maybe due to a coronary artery spasm associated with abnormal course of the coronary vessels, has led us to reduce the possible cardiovascular risks related to the professional activity of the patient.

In fact due to Haskell et al. [22] three major cardiac pathophysiologic processes due to ischemic heart disease in historical and clinical data of the patient may impair his physical working capacity: left ventricular dysfunction, myocardial ischemia and electrophysiologic disturbances as in our patient.

## Conclusion

In conclusion in heart disease worker, the focus of physician is to determine whether or not the increase in cardiac demands produced by physical, psychological and environmental stressors will exceed the threshold for a “safe and sure working capacity” [23,24], especially when there are same anatomical anomaly that con influence this capacity.

## Consent

Written informed consent was obtained from the patient for publication of this Case report and any accompanying images.
